# Transcriptome analysis reveals differential effects of beta-cypermethrin and fipronil insecticides on detoxification mechanisms in *Solenopsis invicta*

**DOI:** 10.3389/fphys.2022.1018731

**Published:** 2022-10-06

**Authors:** Junaid Ali Siddiqui, Yuanyuan Luo, Umer Ayyaz Aslam Sheikh, Bamisope Steve Bamisile, Muhammad Musa Khan, Muhammad Imran, Muhammad Hafeez, Muhammad Imran Ghani, Nie Lei, Yijuan Xu

**Affiliations:** ^1^ Department of Entomology, South China Agricultural University, Guangzhou, China; ^2^ College of Agriculture, College of Tobacco Science, Guizhou University, Guiyang, China; ^3^ Institute for the Control of Agrochemicals, Ministry of Agriculture and Rural Affairs, Beijing, China; ^4^ Department of Entomology, University of Poonch Rawalakot, Rawalakot, Pakistan; ^5^ State Key Laboratory for the Conservation and Utilization of Subtropical Agro-Bioresources, College of Agriculture, South China Agricultural University, Guangzhou, China; ^6^ State Key Laboratory of Rice Biology, Institute of Insect Sciences, Zhejiang University, Hangzhou, China

**Keywords:** alien species, invasive species, ecotoxicology, insecticide resistance, enzymatic detoxification

## Abstract

Insecticide resistance poses many challenges in insect pest control, particularly in the control of destructive pests such as red imported fire ants (*Solenopsis invicta*). In recent years, beta-cypermethrin and fipronil have been extensively used to manage invasive ants, but their effects on resistance development in *S. invicta* are still unknown. To investigate resistance development, *S. invicta* was collected from populations in five different cities in Guangdong, China. The results showed 105.71- and 2.98-fold higher resistance against fipronil and beta-cypermethrin, respectively, in the Guangzhou population. The enzymatic activities of acetylcholinesterase, carboxylases, and glutathione S-transferases significantly increased with increasing beta-cypermethrin and fipronil concentrations. Transcriptomic analysis revealed 117 differentially expressed genes (DEGs) in the BC-ck vs. BC-30 treatments (39 upregulated and 78 downregulated), 109 DEGs in F-ck vs. F-30 (33 upregulated and 76 downregulated), and 499 DEGs in BC-30 vs. F-30 (312 upregulated and 187 downregulated). Kyoto Encyclopedia of Genes and Genomes (KEGG) analysis revealed that DEGs associated with insecticide resistance were significantly enriched in metabolic pathways, the AMPK signaling pathway, the insulin signaling pathway, carbon metabolism, peroxisomes, fatty acid metabolism, drug metabolism enzymes and the metabolism of xenobiotics by cytochrome P450. Furthermore, we found that DEGs important for insecticide detoxification pathways were differentially regulated under both insecticide treatments in *S. invicta*. Comprehensive transcriptomic data confirmed that detoxification enzymes play a significant role in insecticide detoxification and resistance development in *S. invicta* in Guangdong Province. Numerous identified insecticide-related genes, GO terms, and KEGG pathways indicated the resistance of *S. invicta* workers to both insecticides. Importantly, this transcriptome profile variability serves as a starting point for future research on insecticide risk evaluation and the molecular mechanism of insecticide detoxification in invasive red imported fire ants.

## Introduction

Biological invasions continue to threaten ecological systems, species diversity, and ecosystem functions (Simberloff et al., 2013; Pyšek et al., 2020; Cuthbert et al., 2021; Siddiqui et al., 2022a). Exotic species invasion rates are increasing rapidly as a result of global development (Haubrock et al., 2021; Seebens et al., 2021). Invasions have had a wide range of ecological consequences (Dick et al., 2017; Crystal-Ornelas and Lockwood, 2020), including the extinction of indigenous species (Blackburn et al., 2019), reductions in abundance (Bradley et al., 2019), fitness losses (Nunes et al., 2019), changes in ecosystem function, and damage to farmland or human health (Siddiqui et al., 2021). Among these species, the red imported fire ant (RIFA), *Solenopsis invicta* Buren (Hymenoptera: Formicidae), is a global exotic invasive ant species that is considered one of the world’s deadliest invasive species (Huang et al., 2018). *S. invicta* originated in South America and has been introduced worldwide, including to China (Ascunce et al., 2011). RIFA was discovered for the first time in 2003 in Taiwan and then in 2004 in Guangdong Province, later spreading to several areas of China (Zeng et al., 2005). It is designated as a quarantine pest in China because of its invasiveness. RIFA has been demonstrated to negatively affect human health, community safety, the agricultural industry, the environment, and native biodiversity in its invasive range (Vinson, 2013). *Solenopsis invicta* incursions has been shown to threaten 41 animal species on China’s National Protected Wildlife List, including one amphibian, 18 reptiles, and 22 bird species (Vatanparast et al., 2021).

Synthetic insecticides are heavily used to control *S. invicta*, similar to many other insect pests (Williams et al., 2001; Drees et al., 2013). While synthetic insecticides effectively manage ants, public concern has been raised about their adverse effects, including resistance development in targeted insects, environmental degradation, and adverse impacts on the health of humans (Du et al., 2020). In China, many products have already been registered for the control of *S. invicta*. In these products, three main active ingredients, beta-cypermethrin, fipronil, and indoxacarb, are most widely used (Wang et al., 2020a). In contrast to most evolutionary phenomena, resistance has significant practical and economic impacts. In addition to the dramatic increase in the number of resistant species (Siddiqui et al., 2021), the intensity and magnitude of some resistance problems have also increased alarmingly. Resistance to fipronil has evolved in *Blattella germanica* L. (Dictyoptera: Blattellidae) (Holbrook et al., 2003; Gondhalekar and Scharf, 2012). Moreover, the housefly, *Musca domestica*, has developed resistance against beta-cypermethrin (Shi et al., 2020). In line with the well-documented pest resistance resulting from the overuse of insecticides in several insects, it has been reported that such resistance may also develop in RIFA, as these insecticides have been used in RIFA management for numerous years (Morrison and Porter, 2003; Zhang et al., 2016a; Zhang et al., 2016b). In the quest to achieve extensive insect pest management, host resistance analyses are crucial.

Detoxification activity is conferred by significant, advanced systems for insect detoxification or the removal of numerous hazardous chemicals, such as insecticides (Wu et al., 2011). Enzymes responsible for detoxification are involved in various biological processes, functioning against numerous toxins in the body of insects (Lin et al., 2015). In a previous study, the biochemical description of insect tolerance to an insecticide was based on how insensitive the target site was and how metabolic enzymes such as AChE, CarE, and GSTs were able to eliminate the insecticide. These enzymes are responsible for xenobiotic detoxification (Wang et al., 2013; Hu et al., 2014a) and act as biomarkers for assessing resistance, tolerance, and susceptibility (Bues et al., 2005; Khan et al., 2021b). Zhang et al. (2016a) found that fipronil-detoxifying enzymes (cytochrome P450 (CYP) genes) increased RIFA resistance by 36.4%. Another similar investigation revealed that CYP enzymes were critical for the detoxification of fluralaner (Xiong et al., 2020). Moreover, these enzymes help maintain chemical balance and are essential for various physiological functions in insects. Insecticides have been proven to impede the enzymatic balance essential to execute different physiological functions (Chang et al., 2015; Khan et al., 2020). Many studies have indicated that enzyme activity depends on gene expression in all organisms, and a minor change in gene expression can significantly affect enzyme activities (Mao et al., 2019).

The use of Illumina RNA-seq technology for transcriptome analysis in genomic investigations has recently proven to be a reasonable and cost-effective strategy (Lin et al., 2015; Tang et al., 2017). This approach is widely used in diverse insect species to identify the genes involved in reproduction, immunology, metabolism, and insecticide detoxification (Wang et al., 2010; Lin et al., 2015; Liu et al., 2016). Studies of *Cryptolaemus montrouzieri* and *Propylaea japonica* provide two examples of transcriptome-based investigations that have identified insecticide-related genes under conditions of insecticide exposure (He et al., 2012; Tang et al., 2014). The evaluation of the side effects of sublethal insecticide doses may indicate adaptations of biological control agents to sublethal toxic stresses. Additionally, this approach might supply valuable information for improving the insect pest control practices using effective insecticides (Nawaz et al., 2018; Khan et al., 2021a).

The current study examines the mechanism of the sublethal effects of beta-cypermethrin and fipronil on RIFA workers. After the insecticides were applied to different groups of fire ant workers, the activities of AChE, CarE, and GST and transcriptional changes were studied. Our findings will be valuable for further extensive screening investigations of *S. invicta* functional genes involved in insecticide detoxification processes and for highlighting a novel way to understand the molecular mechanisms underlying insecticide stress responses in *S. invicta*.

## Materials and methods

### Insects

Samples of fire ants were collected from localities where insecticides are commonly used across five cities in Guangdong Province: Guangzhou (GZ), Zhongshan (ZS), Zhuhai (ZH), Dongguan (DG), and Jiangmen (JM). Collections were performed in a total of 15 polygyne fire ant colonies these cities. Three colonies from each city were sampled as replications. Fire ant colonies were separated using the water-floating technique, and the ants were placed in plastic containers and maintained in a lab under ambient conditions (26°C and 60% relative humidity) (Chen, 2007; Kafle et al., 2010). To keep the ants from escaping, a talc-ethanol combination was applied to the inner wall of the rearing box (Ning et al., 2019). Food was provided in the form of mealworm larvae (*Tenebrio molitor*) and water tubes containing 10 percent sugar by weight. A few days of acclimatization to the laboratory environment were allotted to the colonies before experimentation so that the ants could perform optimally.

### Insecticides

Beta cypermethrin (95%) (Guangdong Liwei Chemical Co., Ltd) and fipronil (95%) (Anhui Huaxing Chemical Co., Ltd) were used. Beta-cypermethrin and fipronil stock solutions (1000 g/ml) were produced in acetone, and the concentrations were increased according to the geometric ratio (including the control).

### Bioassay

Toxicity bioassays of beta-cypermethrin and fipronil were performed on RIFA workers. The workers’ susceptibility was evaluated using a glass flask residual bioassay to determine the LC10, LC30, LC50, and LC90 of beta-cypermethrin and fipronil (Kanga and Plapp, 1992; Seagraves and McPherson, 2003). Three colonies were examined, and in each colony, three experiments with 30 medium-sized (4–6 mm) worker samples were carried out in a flask under standard circumstances (Chen, 2007).

Acetone was used to produce the stock solutions (1000 g/ml) of insecticides, and it was also used to produce the increasing concentrations according to the geometric ratio (including the control). For each insecticide, ten different concentrations were utilized in bioassays. Each concentration was replicated three times with a total of 30 individuals per iteration. Each flask contained a 1.5 ml vile with a sugar water solution containing 10% sugar. Prior to experimentation, the 50 ml flasks were cleaned with acetone, followed by three separate washes in which detergent and water were used. To prevent the ants from escaping, the flask tops were painted with polytetrafluoroethylene (PTFE). One milliliter of each insecticide-acetone solution was transferred to a glass flask with a glass pipette. The vials were rolled to allow them to dry after adding the solution. After the acetone evaporated, the insecticide residue was evenly distributed inside the vials.

Workers were added to the flasks using a glass rod to avoid injuries. After 24 h, the ants were checked, and the mortality rate was recorded. If an ant did not move after being pricked with a needle, it was considered to be dead. Each experiment included a, acetone control group of ants. The flasks were kept in the laboratory at a temperature of 25 °C and relative humidity of 60%. Following a 24-h insecticide treatment, the surviving RIFA workers were transferred to centrifuge tubes, instantly frozen using liquid nitrogen, and kept at -80 °C for future research.

### Detoxification enzyme activity

The specimens (10–12 workers) exposed to the control and LC30 treatments were weighed prior to homogenization. The RIFA workers were cleaned with 0.05 M sodium phosphate buffer at room temperature (pH 7.3). Homogenized ant samples were centrifuged at 12,000 rpm for 10 min at 4°C. The supernatant was collected, poured into fresh tubes, and centrifuged for 15 min at 12,000 rpm. The final supernatants were utilized for several enzyme analyses. The activity of the detoxifying enzymes (AChE, CarE, and GST) was determined using commercially available kits obtained from the Bioengineering Research Institute of Nanjing Jiancheng, China. We followed the manufacturer’s guidelines. According to the manufacturer’s instructions, a Bio-Rad spectrophotometer (iMark, Japan) was used to measure light absorbance at 412 and 450 nm wavelengths. Furthermore, the protein concentration in the sample was determined using a Bradford assay kit purchased from Beyotime Shanghai, China, and calibrated with bovine serum albumin (BSA) (Khan et al., 2021b).

### RNA sequencing

RNA sequencing was performed on 10–12 workers selected from the population that showed the highest resistance activity; GZ (LC_30_ and control) consisted of three biological replicates. RNA was extracted, and a cDNA library was generated according to the manufacturer’s guidelines using an Illumina HiSeq2500 sample preparation kit (Gene Denovo Biotechnology Co. Guangzhou, China). The quality of the generated raw reads was assessed using QuickP (v 0.18.0) (Chen et al., 2018). The mapping of high-quality filtered reads against the reference genome (http://ftp.ncbi.nlm.nih.gov/genomes/all/GCF/000/789/215/GCF_000789215.1_ASM78921v2) was used for mapping reads by using HISAT2. 2.4 (Kim et al., 2015). The mapped reads were aggregated, and the fragments per kilobase of transcript per million mapped reads (FPKM) value was produced for each transcribed region to assess its variability and richness by using StringTie (version 1.3.1) (Pertea et al., 2015, 2016). The differences in RNA expression between two separate groups and samples were calculated using DESeq2 software (Love et al., 2014) and edgeR (Robinson et al., 2010). Gene Ontology (GO) (Ashburner et al., 2000) and Kyoto Encyclopedia of Genes and Genomes (KEGG) (Ogata et al., 1999; Robinson et al., 2010) pathway analyses were utilized to explore the genetic relationships between DEGs. The raw data were uploaded to the NCBI SRA database (https://www.ncbi.nlm.nih.gov/bioproject/PRJNA876373 accessed on 12 September 2022\x94).

### RT‒qPCR for transcriptomic data validation

Total RNA was obtained using a Tiangen RNA kit and SuperMix, and real-time qualitative RT‒PCR was performed with the EasyScript system (Transgen Biotech, China). The quality and quantity of RNA were determined on a Nanodrop 2000 instrument. RT‒qPCR was performed using Takara Bio Inc. SYBR Premix Ex Taq (Takara Bio Inc.). To perform real-time quantitative RT‒qPCR, we used the Option Real-Time PCR System CFX96 (Bio-Rad, CA). The qPCR program was set to 95°C for 30 s, 136 °C for 5 s, 57°C for 30 s, and 40 cycles for PCR, with Actin serving as an internal reference (Zhao et al., 2009; Lin et al., 2013; Hu et al., 2014b; Clements et al., 2017; Shabbir et al., 2019). In the preliminary experiments, we checked actin expression against the beta-cypermethrin- and fipronil-treated samples and found consistent results. Thus, we used Actin as a reference gene in our study. The corresponding primers were designed in Primer 5 (listed in Supplementary Table S1).

### Statistical analysis

The analysis of data was performed with SPSS (version 22.0). The adjusted mortality was recorded using the Abbott formula (toxicity bioassays) (Abbott, 1925). In probit analysis, the 95% confidence interval was used to calculate LC_10_, LC_30_, and LC_50_ values (CIs). LC_50_ values were considered different when the 95% confidence intervals did not overlap. The following formula was used to compute the resistance ratio:

$$RR=\frac{LC_{50}\;of\;Resistant\;population\;}{LC_{50}\;of\;Susceptible\;population}$$

The results of three replicates of the enzyme activity assays were subjected to a one-way ANOVA followed by the Tukey test to determine the mean variance. Sublethal concentrations and enzyme activities were calculated using R studio. For graphics, Sigma plot version 12.0 was utilized.

## Results

### Toxicity bioassay

The toxicity of beta-cypermethrin and fipronil was explored in the *S. invicta* workers (Table 1). The results of the evaluation of beta-cypermethrin effects against *S. invicta* workers demonstrated the highest computed LC_50_ for the populations collected from GZ 2.95 × 10^−07^ (%), followed by the populations from JM 2.65 × 10^−07^ (%), ZS 2.05 × 10^−07^ (%), ZH 1.68 × 10^−07^ (%), and DG 9.90 × 10^−08^ (%), while the computed LC_50_ of fipronil was highest for the populations collected from GZ 6.00 E^−06^ (%), followed by the populations collected from DG 1.18 × 10^−07^ (%), JM 9.37 × 10^−08^ (%), ZS 6.43 × 10^−08^ (%), and ZH 5.67 × 10^−08^ (%) (Table 1). The evaluation of the variation of the computed LC_50_ in different populations also revealed that in GZ, the resistance against beta-cypermethrin and fipronil in *S. invicta* was high compared to that in the other colonies, while the DG populations were the most susceptible to beta-cypermethrin, and the ZH populations were the most susceptible to fipronil. In addition, the toxicity bioassay results were supplemented by resistance ratio analysis, which showed that fipronil resistance was 105.71 times higher and beta-cypermethrin 2.98-fold higher in the GZ population than in the populations most susceptible to these insecticides.

**TABLE 1:** LC_10_, LC_30_ and LC_50_ values of *Solenopsis invicta* obtained under insecticide exposure.

Insecticide	N	Population	LC_10_ (%) (95%CL)	LC_30_ (%) (95%CL)	LC_50_ (%) (95%CL)	Slope ±se	X2 (df)	RR
Beta-Cypermethrin	2430	GZ	5.66E-10 (1.62E-10-1.54E-09)^a^	2.28E-08 (1.02E-08-4.47E-08)^a^	2.95E-07 (1.63E-07-5.11E-07)^a^	3.081 ± 0.116	11.218 (8)	2.98
	1890	ZS	1.24E-09 (9.30E-11-5.77E-09)^ab^	2.54E-08 (5.39E-09-7.41E-08)^ab^	2.05E-07 (6.99E-08-5.56E-07)^ab^	3.863 ± 0.170	23.331 (6)	2.07
	1890	ZH	8.67E-10 (6.66E-11-4.06E-09)^ab^	1.95E-08 (4.19E-09-5.63E-08)^ab^	1.68E-07 (5.84E-08-4.41E-07)^ab^	3.795 ± 0.168	21.031 (6)	1.70
	1890	DG	3.12E-10 (4.84E-11-1.14E-09)^b^	9.38E-09 (3.03E-09-2.19E-08)^b^	9.90E-08 (4.56E-08-1.98E-07)^b^	3.588 ± 0.164	9.528 (6)	1.00
	2160	JM	4.84E-10 (7.57E-11-1.83E-09)^ab^	2.01E-08 (6.43E-09-4.87E-08)^ab^	2.65E-07 (1.19E-07-5.55E-07)^ab^	3.076 ± 0.127	14.784 (7)	2.68
Fipronil	2160	GZ	1.62E-08 (2.25E-09-6.27E-08)^a^	5.12E-07 (1.53E-07-1.00E-06)^a^	6.00E-06 (2.00E-06-1.30E-05)^a^	2.650 ± 0.108	21.761 (7)	105.71
	1620	ZS	8.13E-10 (8.44E-12-5.35E-09)^ab^	1.07E-08 (7.97E-10-4.18E-08)^ab^	6.43E-08 (1.28E-08-2.53E-07)^ab^	4.856 ± 0.236	26.794 (5)	1.13
	1890	ZH	2.94E-10 (3.34E-11-1.21E-09)^b^	6.59E-09 (1.73E-09-1.70E-08)^b^	5.67E-08 (2.28E-08-1.24E-07)^b^	4.063 ± 0.182	13.424 (6)	1.00
	1890	DG	7.77E-10 (1.38E-10-2.54E-09)^ab^	1.51E-08 (5.22E-09-3.39E-08)^ab^	1.18E-07 (5.53E-08-2.37E-07)^ab^	4.069 ± 0.178	11.865 (6)	2.08
	1890	JM	4.40E-10 (5.02E-11-1.80E-09)^ab^	1.05E-08 (2.78E-09-2.70E-08)^ab^	9.37E-08 (3.78E-08-2.09E-07)^ab^	3.868 ± 0.173	14.15 (6)	1.65

The abbreviations represent the names of the collection areas. ^a, b^ Values are the means of three replicates. The same letter in each row indicates no significant difference across populations at the 5% level. The Kruskal‒Wallis test was performed for columnwise comparisons between the groups at each concentration.

### Activity of detoxification enzymes

**FIGURE 1:**
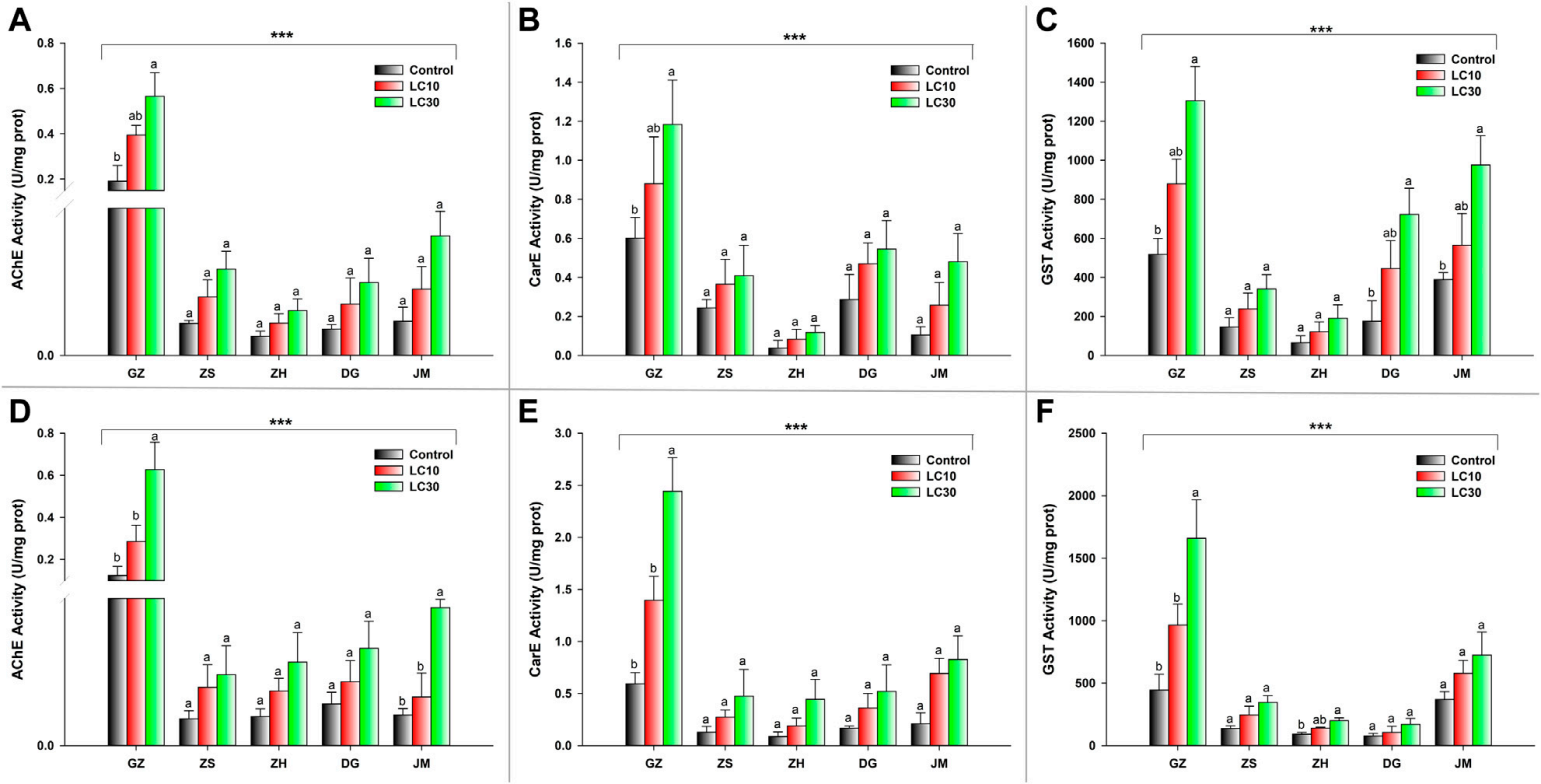
Enzyme activity of **(A)** AChE, **(B)** CarE, and **(C)** GST enzymes in response to beta-cypermethrin and **(D)** AChE, **(E)** CarE, and **(F)** GST enzymes in response to fipronil in the population from GZ, ZS, ZH, DG and JM. The bars represent the average values of three replicates. The standard error of the mean is represented by error bars. The control and two treatments (LC_10_ & LC_30_) were compared using the Tukey HSD all-pairwise comparison test. Bars with the same letters from a specific population showed nonsignificant differences, and asterisks indicate significant differences in enzyme activity between populations (**p < 0.05, ***p < 0.01, Tukey’s test).

The activity of three detoxifying enzymes, AChE, CarE, and GST, was tested in various ant populations exposed to sublethal doses of beta-cypermethrin and fipronil. The results showed that an increase in sublethal doses of beta-cypermethrin and fipronil significantly increased AChE activity across all populations that had been collected. The observed AChE activity in GZ (F_17_ = 6.05, *p* < 0.011, beta-cypermethrin) and (F_17_ = 8.03, *p* < 0.0043, fipronil)) was much higher than that in the populations in other areas (Figures 1A,B). Additionally, CarE activity was much higher in the population that was collected from GZ (F_17_ = 4.24, *p* < 0.034 (beta-cypermethrin); F_17_ = 15.3, *p* < 0.0002 (Fipronil)) than in the other populations. Moreover, the lowest CarE activities against beta-cypermethrin (Figure 1A) and fipronil (Figure 1B) were observed in JM and ZH, respectively. Similarly, GST activity was statistically significantly higher in the population that was obtained from GZ (F_17_ = 8.52, *p* < 0.003 (beta-cypermethrin); (F_17_ = 9.55, *p* < 0.002) (fipronil)) than in the populations collected from other areas. Overall, GST activity was highest in GZ, JM, and DG, but the GZ populations showed higher activity than the ZS and ZH populations (Figure 1C) against beta-cypermethrin, while against fipronil, the JM population showed slightly higher activity than ZS. In comparison, relatively low GST activity was recorded in the populations collected from ZH and DG (Figure 1F). CYP enzymes are also a major constituents of these enzyme suites (Mao et al., 2009). Tolerance to insecticides and the evolution of resistance to insecticides in pest insects rely heavily on CYPs, which play a role in insecticide detoxification. (Lu et al., 2021; Zhao et al., 2022). Previous studies from our university have explored the effects of CYPs on *S. invicta* (Zhang B. et al., 2016; Zhang et al., 2016 B.-Z.), so we focused on the other enzymes to study their effects on *S. invicta*.

### Transcriptional analysis of beta-cypermethrin- and fipronil-treated *S. invicta*

Transcriptional analysis of *S. invicta* samples under treatment with two insecticides was performed. A total of 48.12–51.2 (BC-ck), 44.92–50.96 (BC-30), 50.14–58.41 (F-ck), and 46.92–53.49 (F-30) million raw reads were obtained after treatment with the insecticides beta-cypermethrin (BC) and fipronil (F), respectively (Supplementary Table S2). Among the total clean reads from the six samples, Q30 values of 93.46–95.04% and GC contents of 36.16–44.16% were obtained by a quality control process (Supplementary Table S3). Most of the clean reads (99.61–99.85%) from all libraries were mapped to the reference genome according to the alignment results (Supplementary Table S3). The saturation curve indicated that genes with modest expression were also saturated. This suggests that the collected reads were adequate to secure the large percentage of the expressed genes. The curves used in the gene coverage study were not skewed. The results showed that the reference genome’s data were distributed uniformly among its genes. The Illumina sequencing results were found to be of high quality in this investigation.

### Differentially expressed genes analysis of *S. invicta* under treatment with two insecticides

**FIGURE 2:**
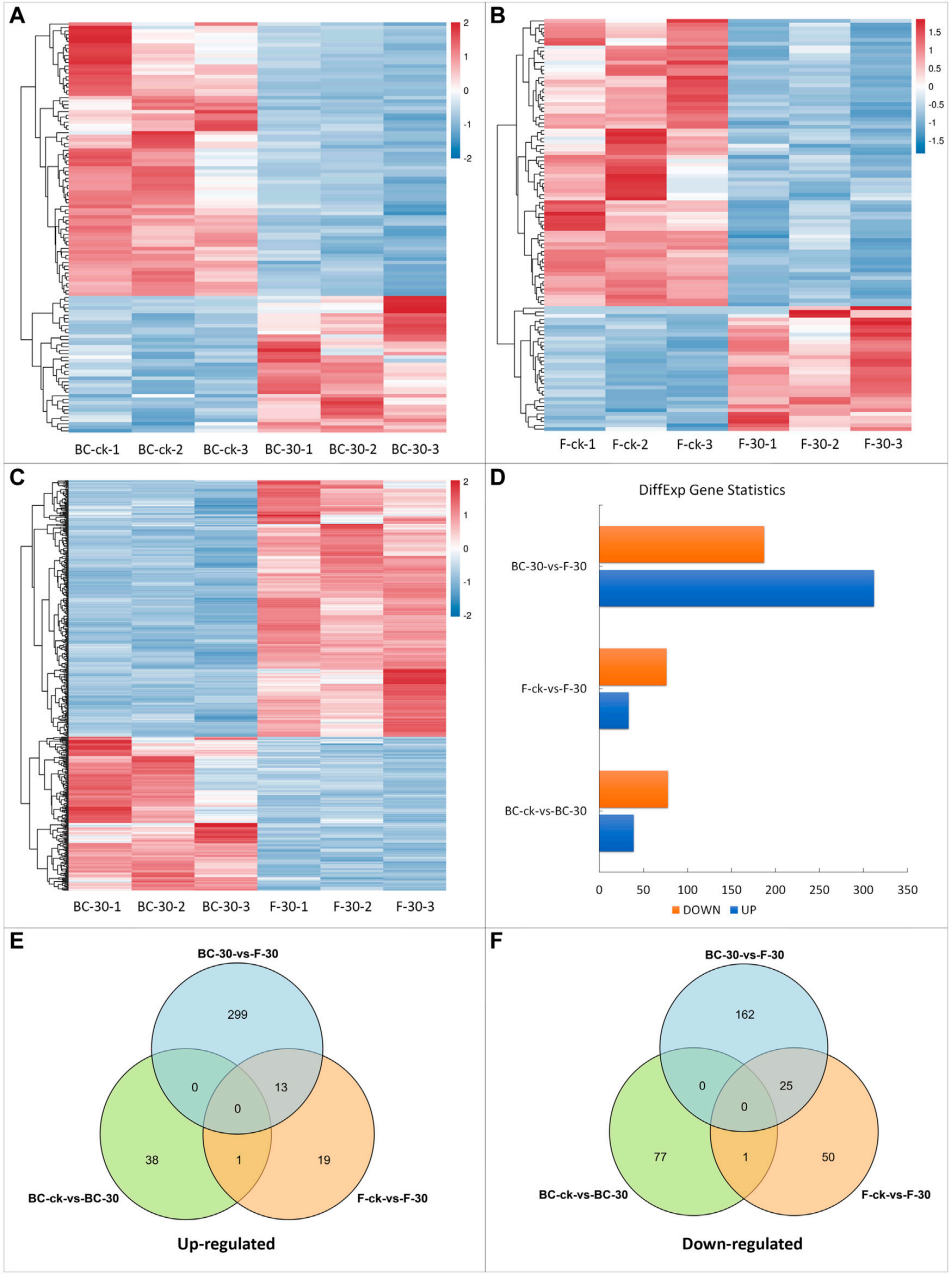
DEGs in response to insecticide treatments. **(A)** DEGs of BC-ck vs. BC-30, **(B)** DEGs of F-ck vs. F-30, **(C)** DEGs of BC-30 vs. F-30, **(D)** up- and downregulated genes among DEGs, **(E)** Venn diagram of upregulated DEGs from all treatments, and **(F)** Venn diagram of downregulated DEGs.

To identify statistically significant DEGs, we compared the expression levels of each gene in control libraries in a pairwise manner and filtered the results according to a log2 (fold change) ≥ 1 and FDR <0.05. Overall, 725 DEGs were identified among all libraries (Figure 2). Pairwise comparative analysis (BS–ck vs. BC–30, F–ck vs. F–30, BC–30 vs. F–30) of the DEGs was performed to thoroughly investigate the possible molecular mechanism of insecticide detoxification in fire ants. Among 725 DEGs, 16% in BS–ck vs. BC–30, 15% in F–ck vs. F–30, and 68% in BC–30 vs. F–30 were significantly regulated. In total, 117 DEGs (16%) (39 upregulated and 78 downregulated) were found in BC–ck vs. BC–30 (Figure 2D, Supplementary Figure S1A), and 109 (15%) DEGs (33 upregulated and 76 downregulated) were found in F–ck vs. F–30 (Figure 2D, Supplementary Figure S1B). Moreover, 499 (68%) DEGs (312 upregulated and 187 downregulated) were found in BC–30 vs. F–30 (Figure 2D, Supplementary Figure S1C).

In summary, these findings implied that there was statistically significant variation in gene expression levels after both insecticide treatments in fire ants. Furthermore, the total numbers of DEGs that were upregulated and downregulated in each comparison and their overlap are presented in Venn diagrams (Figures 2E,F). Overall, a total of 14 upregulated genes (Figure 2E) and 26 downregulated genes (Figure 2F) overlapped. Moreover, no overlapping genes were detected among the BC–ck vs. BC–30, F–ck vs. F–30, and BC–30 vs. F–30 comparisons (Figures 2E,F).

Additionally, principal component analysis (PCA) grouped BC-30, F-ck and F-30 into separate quadrants, while two replications of BC-ck shared the same quadrant as F-ck (Supplementary Figure S2A). Almost all groups showed a strong correlation within the same sample and significant variation among the different treatments, which revealed a strong correlation (Supplementary Figure S2A). A heatmap of all DEGs from a particular time point is shown in Supplementary Figure S2B.

### GO and KEGG enrichment analysis of differentially expressed genes

**FIGURE 3:**
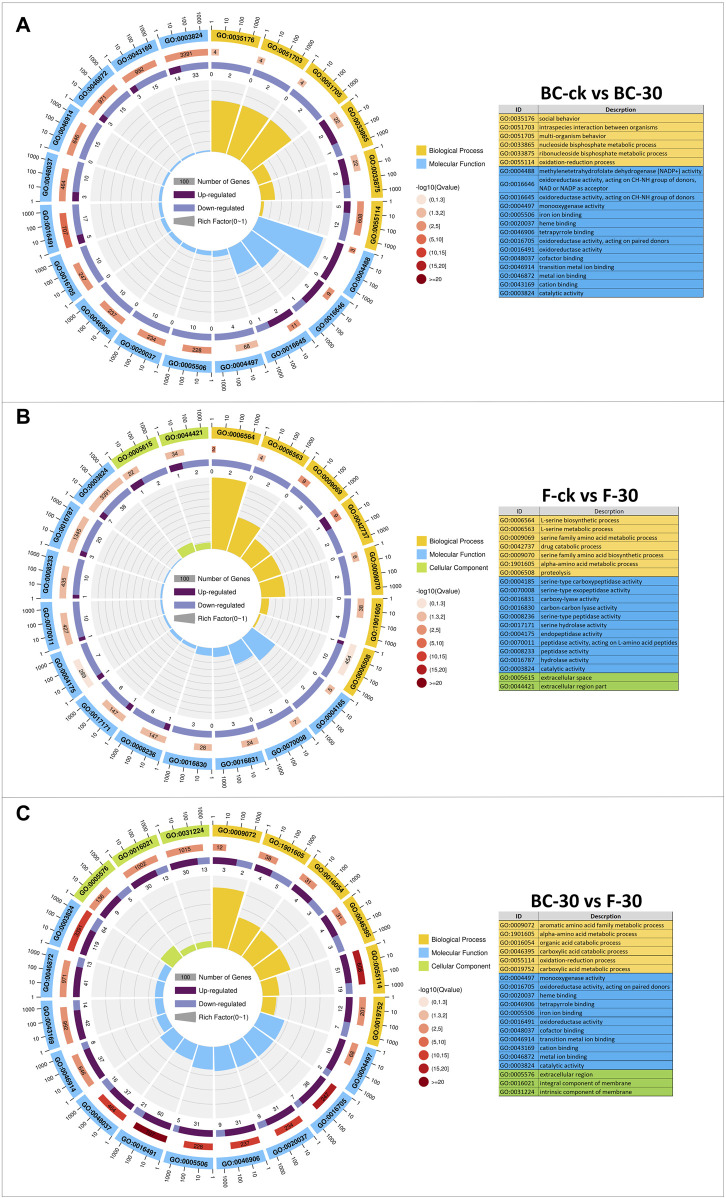
GO classifications **(A)** (BC-ck vs. BC-30 **(B)** F-ck vs. F-30, and **(C)** BC-30 vs. F-30. Biological processes, cellular components, and molecular functions are indicated in different colors.

A GO-based enrichment approach was applied to identify the functions of the DEGs, and these DEGs were distributed into the biological process (BP), molecular function (MF), and cellular component (CC) functional categories (Figure 3, Supplementary Table S4). In the BC–ck vs. BC–30 comparisons, 117 (51.7%) DEGs showed GO annotations, and most of them were substantially enriched in 24 functional categories, including biological process terms (GO:003576, GO:0051703, GO:0051705, GO:0033865, GO:0033875, GO:0055114) and molecular function terms (GO:0004488 GO:0016646, GO:0016645, GO:0004497, GO:0005506, GO:0020037, GO:0046906, GO:0016705, GO:0016491, GO:0048037, GO:0046914, GO:0046872, GO:0043169, GO:0003824) (Figure 3A). In the F-ck vs. F-30 comparison, 109 (48.2%) DEGs showed GO annotation, and most of them were enriched in 27 subgroups, including biological process terms (GO:0006564, GO:0006563, GO:0009069, GO:0042737, GO:0009070, GO:1901605, GO:0006508), molecular function terms (GO:0004185, GO:0070008, GO:0016831, GO:0016830, GO:0008236, GO:0017171, GO:0004175, GO:0070011, GO:0008233, GO:0016787, GO:0003824), and cellular component terms (GO:0005615, GO:0044421) (Figure 3B). In the BC–30 vs. F–30 comparisons, all the DEGs showed GO annotation, and most of them were enriched in 39 subgroups, including biological process terms (GO:0009072, GO:1901605, GO:00165054, GO:0046395, GO:0055114, GO:0019752), molecular function terms (GO:0004497, GO:0016705, GO:0020037, GO:0046906, GO:0005506, GO:0016491, GO:0048037, GO:0046914, GO:0043169, GO:0046872, GO:0003824) and cellular component terms (GO:0005576, GO:0016021, GO:0031224) (Figure 3C). Surprisingly, DEGs associated with biological processes, cellular components, and molecular functions were downregulated in the BC-ck vs. BC-30 and F-ck vs. F-30 comparisons, while in BC–30 vs. F-30, the maximal number of DEGs were upregulated. Furthermore, the biological process category showed the most GO annotations, followed by cellular components and molecular functions.

**FIGURE 4:**
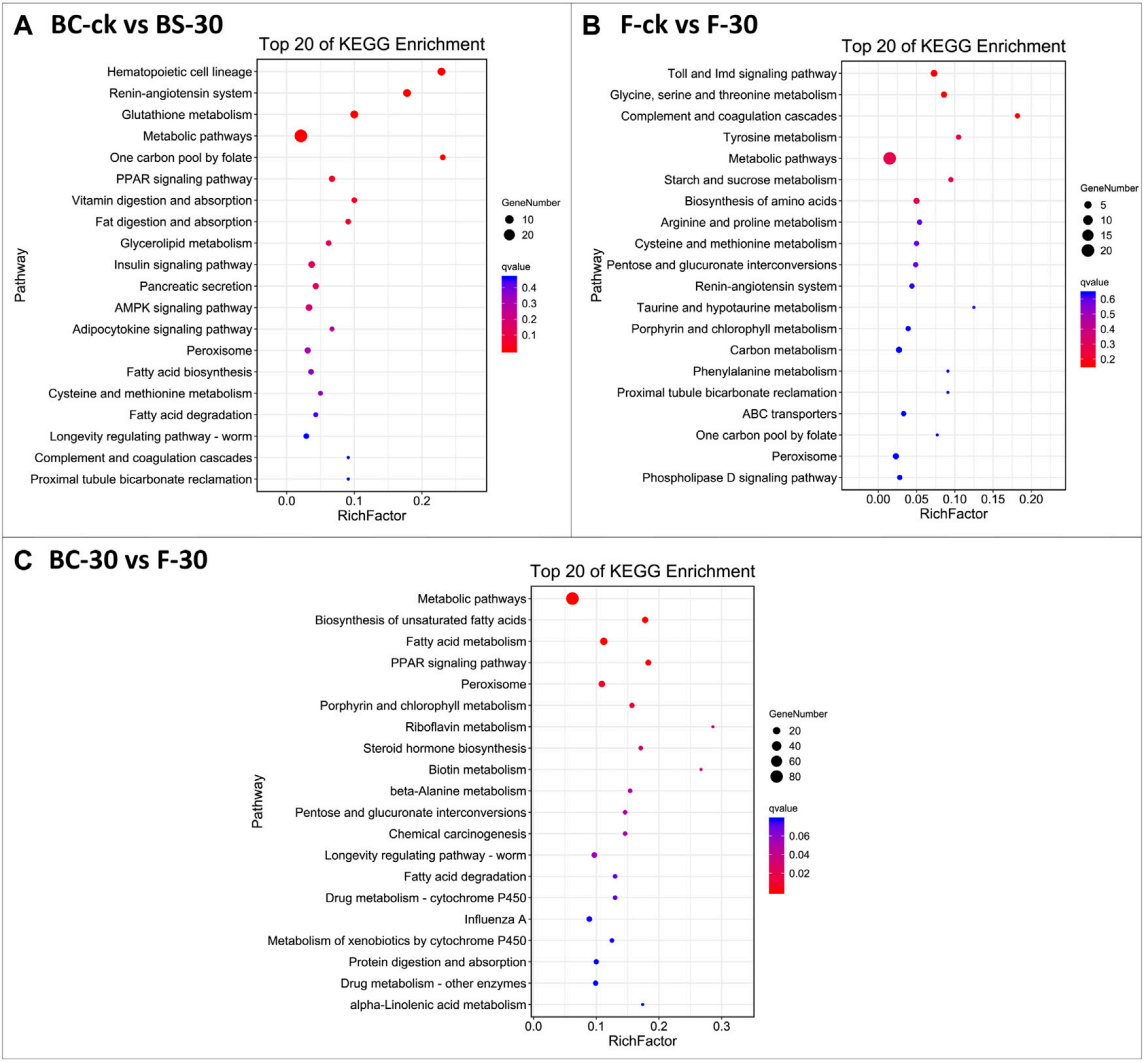
The most enriched Kyoto Encyclopedia of Genes and Genomes (KEGG) pathways of *S. invicta* **(A)** BC-ck vs. BC-30 **(B)** F-ck vs. F-30, and **(C)** BC-30 vs. F-30. The significance of pathways is indicated by the q-value (color bar), the rich factor (X-axis), and the circles indicating the numbers of differentially regulated genes.

The KEGG database was used to classify DEGs based on the pathways or functions to which they contribute. We integrated the annotated sequences into the KEGG database to determine which pathways were activated in the fire ants after insecticide application. Our KEGG pathway enrichment analysis results revealed some common pathways between comparisons performed under the two insecticide treatments (Figure 4, Supplementary Table S5). Among them, metabolic pathways, the insulin signaling pathway, the AMPK signaling pathway, and peroxisomes were significantly enriched KEGG pathways in BC-ck vs. BC-30 (Figure 4A); metabolic pathways, carbon metabolism and peroxisomes were considerably enriched KEGG pathways in F-ck vs. F-30 (Figure 4B); and metabolic pathways, fatty acid metabolism, and peroxisomes were the most enriched KEGG pathways in the BC-30 vs. F–30 comparisons (Figure 4C). In addition, two unique significantly enriched pathways, metabolism of xenobiotics by cytochrome P450 and drug metabolism–other enzyme, were found only in the BC-30 vs. F-30 comparisons.

### Analysis of putative genes involved in the regulatory mechanism of insecticide detoxification

KEGG annotation was used to investigate the putative insecticide detoxification genes in *S. invicta* populations to better understand the detoxification pathway and reveal the genes involved. Previously, it was reported that detoxification involves enzymes that catalyze sequential reactions to aid in the reduction of the toxicity of the insecticidal molecules and more effectively excrete them from the body. Acetylcholinesterase (AChE), carboxylstrease (CarE) and glutathione S-transferases (GST) are important enzymes that catalyze the oxidation, hydrolysis and reduction of toxins (Hilliou et al., 2021). The roles of detoxifying enzymes in the insecticide metabolism have been further investigated in Acari species. For instance, the two-spotted spider mite (*Tetranychus urticae)*, a phytoseiid (*Phytoseiulus persimilis),* the European red mite (*Panonychus ulmi)*, a hard tick (*Rhipicephalus bursa),* and the scabies mite (*Sarcoptes scabiei)* show increases in the expression or activities of CCEs, GSTs, and CYPs and are resistant to insecticides such as abamectin, etoxazole, tebufenpyrad, pyrethroids, and spirodiclofen (Wu and Hoy, 2016).

**FIGURE 5:**
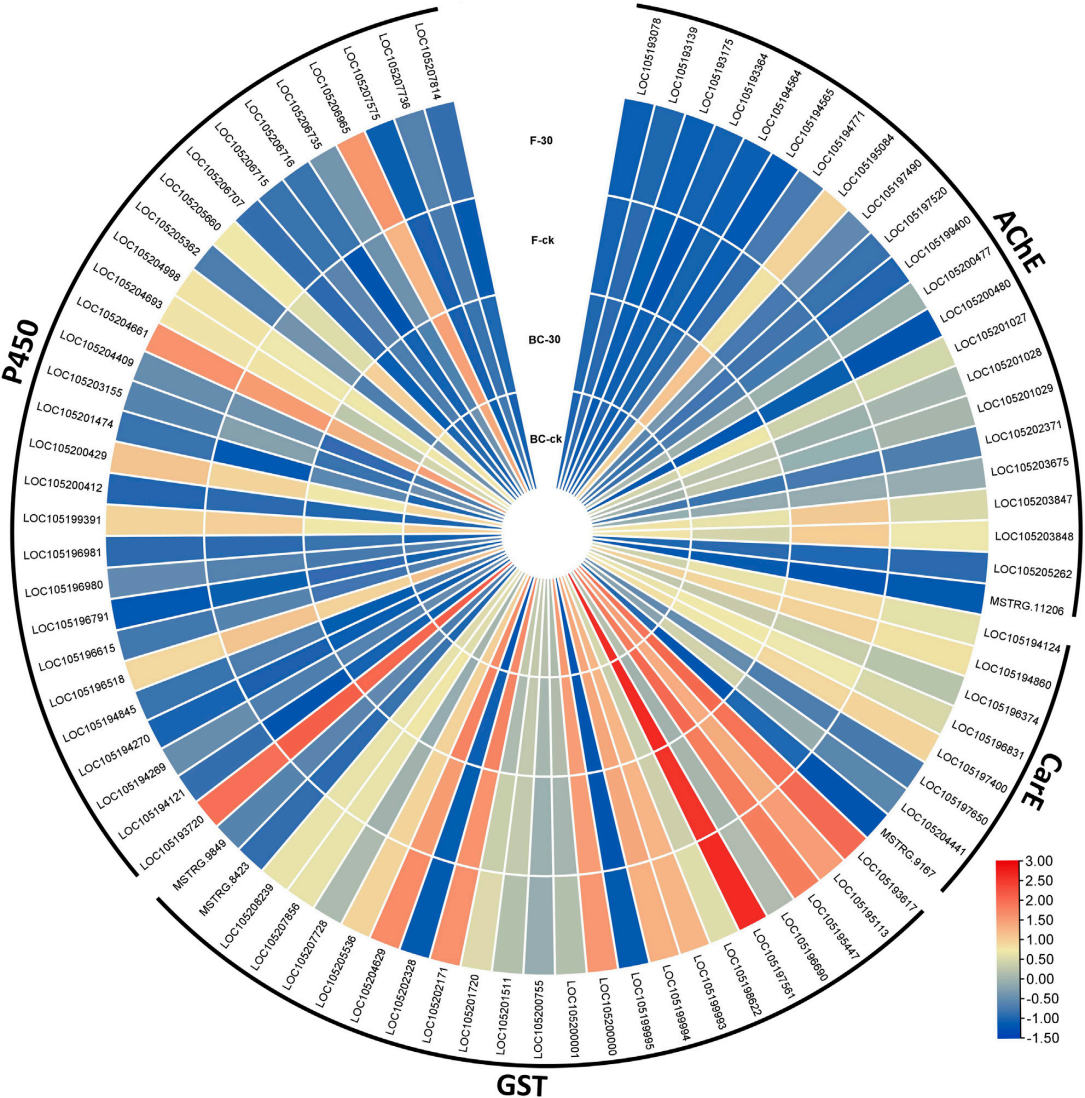
Comparison of significantly regulated insecticide detoxification genes in BC-ck vs. BC-30, F-ck vs. F-30 and BC-30 vs. F-30.

Therefore, considering the key enzymes and their KEGG pathways associated with the detoxification of insecticides, we screened the DEGs related to acetylcholinesterase, carboxylesterase, glutathione S-transferases and cytochrome-P450 identified in the BC-ck vs. BC-30 and F-ck vs. F-30 comparisons. A total of 82 DEGs related to the insecticide detoxification mechanism were identified and presented in a heatmap (Figure 5). Furthermore, 22 genes related to AChE, eight genes related to CarE, 23 genes related to GSTs and 29 genes associated with CYPs were found (Figure 5, Supplementary Tables S6&S7).

**FIGURE 6:**
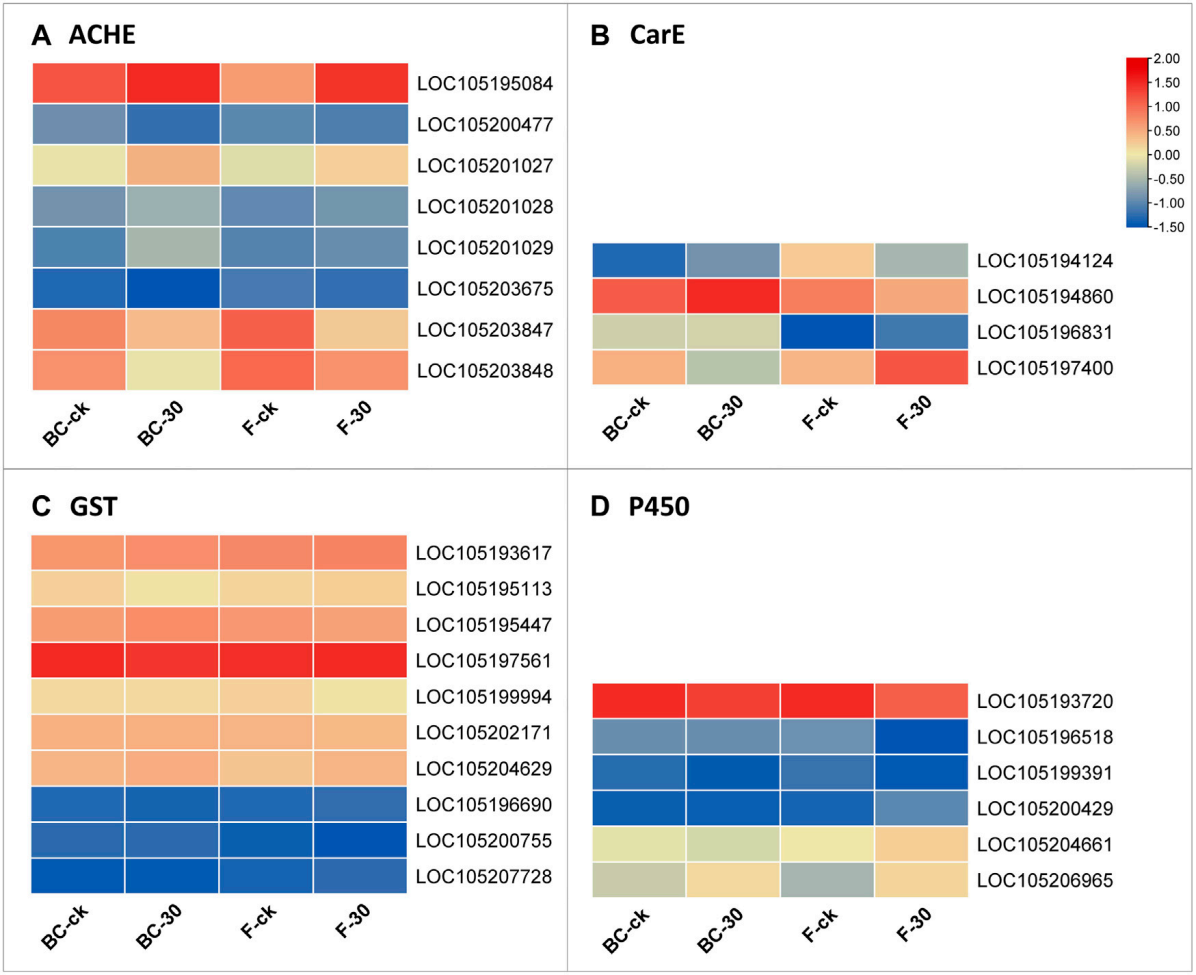
Genes significantly involved in insecticide detoxification according to the analysis of related **(A)** ACHE, **(B)** CarE, **(C)** GST, and **(D)** P450 enzymes.

We further screened the total DEGs to narrow down the detoxification pathways and identify the crucial genes involved in the molecular regulatory mechanism of insecticide metabolism. Among the 82 genes from BC-ck vs. BC-30 and F-ck vs. F-30, we selected 22 key regulatory pathway genes, including *LOC105195084* (acetylcholine receptor subunit alpha-like 1, transcript variant X1), *LOC105200477* (acetylcholine receptor subunit alpha-L1), *LOC105201027* (acetylcholine receptor subunit alpha-type acr-16-like), *LOC105201028* (neuronal acetylcholine receptor subunit alpha-3-like), *LOC105201029* (acetylcholine receptor subunit alpha-like 1, transcript variant X2), *LOC105203675* (acetylcholine receptor subunit beta-like 2), *LOC105203847* (neuronal acetylcholine receptor subunit alpha-2-like), *LOC105203848* (neuronal acetylcholine receptor subunit alpha-2-like) from acetylcholinesterase (Figure 6A), *LOC105194124* (venom carboxylesterase-6-like), *LOC105194860* (venom carboxylesterase-6-like), *LOC105196831* (liver carboxylesterase-like), *LOC105197400* (venom carboxylesterase-6, transcript variant X1) from Carboxylesterase (Figure 6B), *LOC105193617* (probable glutathione peroxidase 2), *LOC105195113* (microsomal glutathione S-transferase 1-like), *LOC105195447* (glutathione S-transferase theta-1-like, transcript variant X5), *LOC105197561* (glutathione S-transferase-like), *LOC105199994* (glutathione S-transferase-like), *LOC105202171* (probable phospholipid hydroperoxide glutathione peroxidase, transcript variant X2), *LOC105204629* (lactoylglutathione lyase, transcript variant X2), *LOC105196690* (glutathione S-transferase C-terminal domain-containing protein homolog), *LOC105200755* (glutathione synthetase-like), and *LOC105207728* (glutathione synthetase-like) from the Glutathione S-transferase family (Figure 6C), which were differentially expressed under both treatments.

Furthermore, we surveyed the cytochrome-P450 (CYP) pathway to identify detoxification genes that were differentially expressed in the regulatory mechanism of insecticides. Among the cytochrome-P450 pathways, *LOC105193720* (cytochrome P450 4g15-like), *LOC105196518* (cytochrome P450 4C1-like), *LOC105199391* (cytochrome P450 CYP12A2-like), *LOC105200429* (XP_025988557.1 probable cytochrome P450 304a1), *LOC105204661* (cytochrome P450 6j1-like), and *LOC105206965* (cytochrome P450 4C1-like) were the mainly genes included in the upregulated genes (Figure 6D). Overall, these findings suggest interconnections between the above pathways regulating insecticide detoxification mechanisms in *S. invicta*.

### Validation of insecticide detoxification genes

**FIGURE 7:**
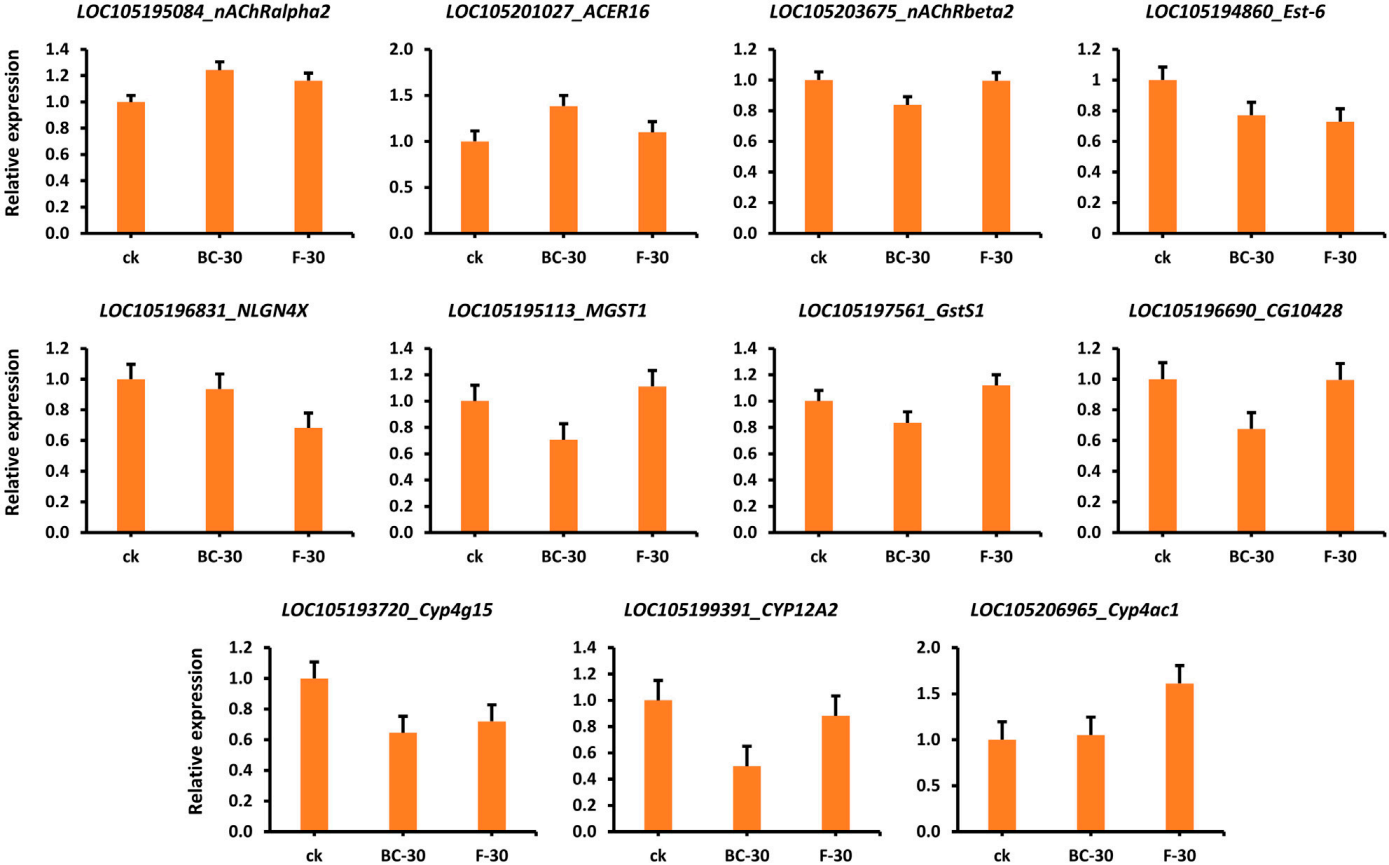
Validation of several related DEGs involved in the detoxification of beta-cypermethrin and fipronil in *Solenopsis invicta* through qPCR.

To validate the obtained expression profiles, RT–qPCR was used to quantify genes involved in the regulatory mechanisms of insecticide detoxification (i.e., HPGDS, cat2, nAChRbeta1, ARA1, ache, acD, Est-5A, nAChRalpha1, D11DS, desat1, SCD, CES2, and ACBD5) that were chosen from the RNA-seq data (Supplementary Table S1, Figure S3A,B). The RNA-seq-based relative gene expression information is shown in Figure 7. For example, the *nAChRalpha2*, *ACER16* and *Cyp4ac1* genes showed significantly higher expression in the control than in the BC-30 and F-30 treatments. In contrast, the expression levels of *Est-6, NLGN4X, Cyp4g15, and CYP12A2* were downregulated in the control compared with the treatments. Furthermore, the *MGST1* and *GstS1* genes showed higher expression in the F-30 treatments than in the control, and BC-30 was involved in the mechanism of insecticide detoxification. Overall, various genes displayed differential responses of up- or downregulation compared with the control in the molecular mechanism of insecticide detoxification. These results suggest that the data obtained by RNA-seq are reliable and capable of being reproduced when compared to data obtained from other sources, such as RT‒qPCR (Supplementary Table S1, Figure S3A,B).

## Discussion

Invasive ants are considered highly devastating in regions where they were initially introduced (Holway et al., 2002). In numerous cases, ant invasions have led to declines and extinctions of a range of native taxa, particularly ants, as a result of direct interactions *via* predation or indirect interactions *via* exploitative competition for available food resources (Lach and Hooper-Bui, 2010; Bertelsmeier et al., 2015). Invasive ants include many aggressive species of ants that displace native species through competition and predation. The abundance and diversity of indigenous ant populations are reduced mainly due to exploitation and competition with *S. invicta* (Porter and Savignano, 1990).

Synthetic insecticides are a relatively reliable, effective and rapid tool for managing pest problems (Khan et al., 2018, 2020; 2021b). However, there are numerous problems associated with unmonitored and unplanned insecticide applications (e.g., adverse effects on the environment and harmful effects on nontarget organisms, such as humans, land and aquatic animals, and other arthropods), and the most important problem is the development of resistance (Nawaz et al., 2017; Rasheed et al., 2020; Liu et al., 2021). Increased insecticide resistance imposes challenges on the sustainable management of many insect infestations (Bass and Jones, 2018). Pyrethroids are among the most widely used insecticides against *S. invicta* in China (Wang et al., 2020a). On the other hand, the repeated and heavy use of these compounds has led to the rapid development of *S. invicta* resistance (Wang et al., 2020b).

In the current study, beta-cypermethrin and fipronil were likewise effective against the *S. invicta* populations collected from GZ, ZS, ZH, DG, and JM. Buczkowski and Wossler (2019) reported a significant reduction in an Argentine ant, *Linepithema humile* Mayr, population after applying fipronil. Their study showed that even small quantities of fipronil cause primary and secondary mortality in Argentine ants. Collins and Callcott (Collins and Callcott, 1998) also reported that fipronil could be employed as an ultralow-dose bait for *S. invicta*. A 15 μg/mg dose was an effective granular bait for achieving 80% mortality within 2–12 weeks. However, in the current study, the *S. invicta* populations collected from GZ showed a high resistance ratio (105.71-fold higher) against fipronil than the population collected from ZS, which was most susceptible to fipronil. Kafle and Shih (2012) reported that 0.13% cypermethrin mixed with distiller’s dried grains with solubles (DDGS) easily caused 50% mortality in fire ants. The current study results revealed that beta-cypermethrin is still an effective insecticide against fire ants, with only a 2-fold increase in resistance. The resistance ratios of the *S. invicta* populations collected from ZS, ZH, DG and JM also indicated less resistance against beta-cypermethrin than was observed in GZ. Moreover, the resistance ratios of the GZ and JM populations against beta-cypermethrin were higher than those of other populations collected from ZS, ZH, and DG.

Insecticides at sublethal concentrations can have behavioral and physiological impacts on target pests (Desneux et al., 2007), resulting in amplified resistance against these insecticides. This could be one of the reasons for pest resurgence, and insect pests have the potential to adapt rapidly to insecticide selection by metabolizing, neutralizing, or detoxifying insecticides (Dawkar et al., 2016). Several studies have been conducted to determine the correlations between enzyme activity and insecticide resistance (Wu et al., 2014; Zhang et al., 2016a; Zhang et al., 2016b; Tang et al., 2017; Xiong et al., 2019). Enzyme activity is often employed as an efficient indicator in monitoring insects for insecticide resistance development. Various detoxifying xenobiotics function as detoxification enzymes in insects, including AChE, CarE, GST, and CYPs, which are responsible for resistance development. Because ants regularly contact toxins, high detoxification enzyme activity must be critical to their survival (Chen et al., 2014; Zhang et al., 2016a; Zhang et al., 2016b; Siddiqui et al., 2022b).

Carboxylesterase and acetylcholinesterase are members of the esterase family of enzymes, which enable insects to detoxify insecticides. The present study showed that the activity of AChE and CarE increased with an increase in the insecticide concentration. The activity of AChE and CarE was higher in resistant populations than in susceptible populations. In previous investigations, fipronil has been shown to increase the activity of CarE in honeybees (Carvalho et al., 2013; Roat et al., 2017). Jin et al. (2020) reported that the activity of CarE was much higher in a resistant strain of the grassland locusts *Epacromius coerulipes* Ivanov (Orthoptera: Acrididae) than in a susceptible strain. Alpha and beta esterase activity was higher in a resistant strain of the mosquito *Culex quinquefasciatus* Say (Diptera: Culicidae) than in a susceptible strain when exposed to fipronil (Sarkar et al., 2009). The beta-cypermethrin exposure of a resistant strain of the house fly, *Musca domestica* Linnaeus (Diptera: Muscidae), resulted in higher activity of CarE than was observed in a beta-cypermethrin-susceptible strain (Zhang et al., 2007). This insecticide has been shown to increase honey bee CarE activity in previous investigations (Carvalho et al., 2013; Roat et al., 2017; Xiong et al., 2019). The enzymes most frequently reported to be involved in the detoxification of penetrating xenobiotics are general esterases (ESTs) and glutathione S-transferases (GST) (Zibaee et al., 2008). Glutathione transferases (GSTs) are a large and diverse family of enzymes found in virtually all aerobic organisms. They play essential roles in detoxifying both endogenous and xenobiotic chemicals and in intracellular transportation, hormone production, and oxidative stress protection (Enayati et al., 2005). The current study demonstrates a significant change in GST activity due to exposure to beta-cypermethrin and fipronil. The results also show that sublethal concentrations and population resistance have a significant impact on GST activity. Zhang et al. (2007) found that GST activity was also significantly increased in a resistant strain of *M. domestica*. Sarkar et al., 2009) observed that the GST activity of all examined populations was higher than that of a susceptible laboratory strain. A resistant strain of *E. coerulipes* showed significantly higher GST activity than susceptible insects (Jin et al., 2020). Another previous study also indicated an increase in GST activity following the application of an insecticide against corn rootworm (Scharf and Siegfried, 1999).

Insects have developed various complex detoxification mechanisms to survive exposure to a wide range of poisonous substances. For example, detoxification enzymes are crucial for removing these harmful chemicals from the body (Wu et al., 2011). It is well established that insecticides disrupt the enzymatic equilibrium required to carry out several physiological activities (Ostrowski et al., 2002; Gong et al., 2013; Chang et al., 2015; Khan et al., 2021b). Many detoxification-related pathways can be deployed to cope with insecticide toxicities in living organisms, such as xenobiotic metabolism by cytochrome P450 enzymes, steroid hormone biosynthesis, glycerophospholipid metabolism, glutathione metabolism, retinol metabolism, chemical carcinogenesis, drug metabolism, and linoleic acid metabolism. Poisonous lipophilic material is combined with hydrophilic molecules in the xenobiotic metabolic pathway to rapidly eliminated this material from living organisms (Faber et al., 2014). Our study showed that fipronil and beta-cypermethrin regulated several genes related to detoxification. Among these genes, 82 genes related to detoxification enzymes were found to be significantly differentially regulated, revealing that *S. invicta* insecticide exposure triggered the deployment of detoxification enzymes, which might be the key factor in insecticide resistance in *S. invicta.* Moreover, different detoxification pathways can be used as biomarkers to assess the effects of a specific insecticide on an organism. Khan et al. (2021a) reported many detoxification-related pathways in insects that play key roles in gene regulation during insect host exposure to insecticides. The current study also revealed many detoxification pathways (e.g., metabolic pathways, the insulin signaling pathway, the AMPK signaling pathway, carbon metabolism and peroxisomes, fatty acid metabolism, the metabolism of xenobiotics by cytochrome P450 and the drug metabolism–enzyme pathway) that were significantly differentially regulated due to the exposure of *S. invicta* to fipronil and beta-cypermethrin.

Most xenobiotic chemicals are lipophilic, making it challenging for the body to eliminate them (Faber et al., 2014). As a result, these lipophilic chemicals tend to concentrate in living organisms, frequently causing detrimental impacts on biological processes. Hence, lipophilic chemicals are converted to hydrophilic compounds through xenobiotic metabolism, making them easier for living organisms to eliminate (Nawaz et al., 2018). However, multiple investigations have shown that these metabolites produced during xenobiotic digestion may occasionally be more toxic than the insecticidal agents themselves (Laine et al., 2009; Grilo et al., 2014; Nawaz et al., 2018). These insecticides trigger the expression of detoxification enzymes and related genes in the body of living organisms. Previous studies have shown that exposure to beta-cypermethrin and fipronil significantly regulates genes related to the detoxification of insecticides (Zhang et al., 2016a; Hilliou et al., 2021). For example, exposure to beta-cypermethrin significantly upregulated carboxylesterase and GST-related genes, while several genes were downregulated from quantities of one to zero in *Culex pipiens* (Li et al., 2016). In contrast, Xiao et al. (2017) reported that beta-cypermethrin exposure arrested the normal regulation of esterase and GST-related genes. The results showed that the number of upregulated genes in *Harmonia axyridis* was lower (868 DEGs) than that of downregulated genes (2,248 DEGs). The gene expression of GST-related genes was shown to be upregulated *via* RT‒qPCR in *Heortia vitessoides* following exposure to beta-cypermethrin (Cheng et al., 2018). A related study in which fipronil was used to treat *Epacromius coerulipes* showed that 52 carboxylesterase and 25 GST-related genes were significantly up- or downregulated. Gene expression was found to be higher in a susceptible strain of *E. coerulipes* than in a resistant strain (Jin et al., 2020). In the current study, the expression of detoxification genes dramatically changed following exposure to the two pesticides, indicating that treatment with the two insecticides significantly impacted insect metabolism, similar to findings of previous studies (Zhang et al., 2016a; Hilliou et al., 2021). Moreover, the number of downregulated genes was greater than the number of upregulated genes between the BC-ck vs. BC-30 and F-ck vs. F-30 treatments, and the same pattern was noted by Xiao et al. (2017). While more upregulated genes were observed in BC-30 vs. F-30, these results were supported by the findings of Li et al. (2016). There were more upregulated genes in the fipronil-exposed group than in the beta-cypermethrin group, which also confirmed the speculation from earlier studies that *S. invicta* is more resistant to fipronil than to beta-cypermethrin (Wang et al., 2020a). Some patterns of transcriptional changes observed in this study were different from those in previous studies because of the different insects used. In the case of fire ants, further studies are needed to better understand insecticide resistance mechanisms.

In general, little is currently known about the potential insecticide-detoxifying pathways of *S. invicta*. However, in this study, we successfully identified highly expressed genes that play important roles in *S. invicta* resistance against insecticide treatment. In addition, these findings show that detoxification enzymes (AChE, CarE, GST, and CYPs) may influence the detoxification of insecticides in *S. invicta*. However, more research is needed to determine their precise functions in the metabolism of these insecticides.

## Conclusion

The current study examined resistance against fipronil and beta-cypermethrin in *S. invicta*. Red imported fire ants were shown to have developed 105.71-fold resistance against fipronil and 2.98-fold resistance against beta-cypermethrin. The observed resistance against different insecticides was higher in ants from the vicinity of Guangzhou than in other cities of Guangdong Province. AChE, CarE, GST, and CYP enzymes were significantly involved in fipronil and beta-cypermethrin resistance. These results imply that red imported fire ants have already developed resistance against fipronil. Thus, the application of this insecticide should be immediately stopped, and it should be replaced with chemical compounds to which the ants do not show resistance, such as beta-cypermethrin. Numerous insecticide-related genes, GO terms, and KEGG pathways that were identified indicated the resistance of *S. invicta* workers to both insecticides. Importantly, this transcriptome profile variability serves as a starting point for future research on insecticide risk evaluation and the molecular mechanism of insecticide detoxification in invasive red imported fire ants.

## Data Availability

The original contributions presented in the study are included in the article/Supplementary Material, further inquiries can be directed to the corresponding authors.
