# Supplementary material for: Transcriptome analysis reveals differential effects of beta-cypermethrin and fipronil insecticides on detoxification mechanisms in Solenopsis invicta
Source: Front Physiol. 2022 Oct 6;13:1018731. doi: 10.3389/fphys.2022.1018731 (PMC9583148; doi:10.3389/fphys.2022.1018731)
Supplement: Supplementary file 1 [file DataSheet2.docx]

**
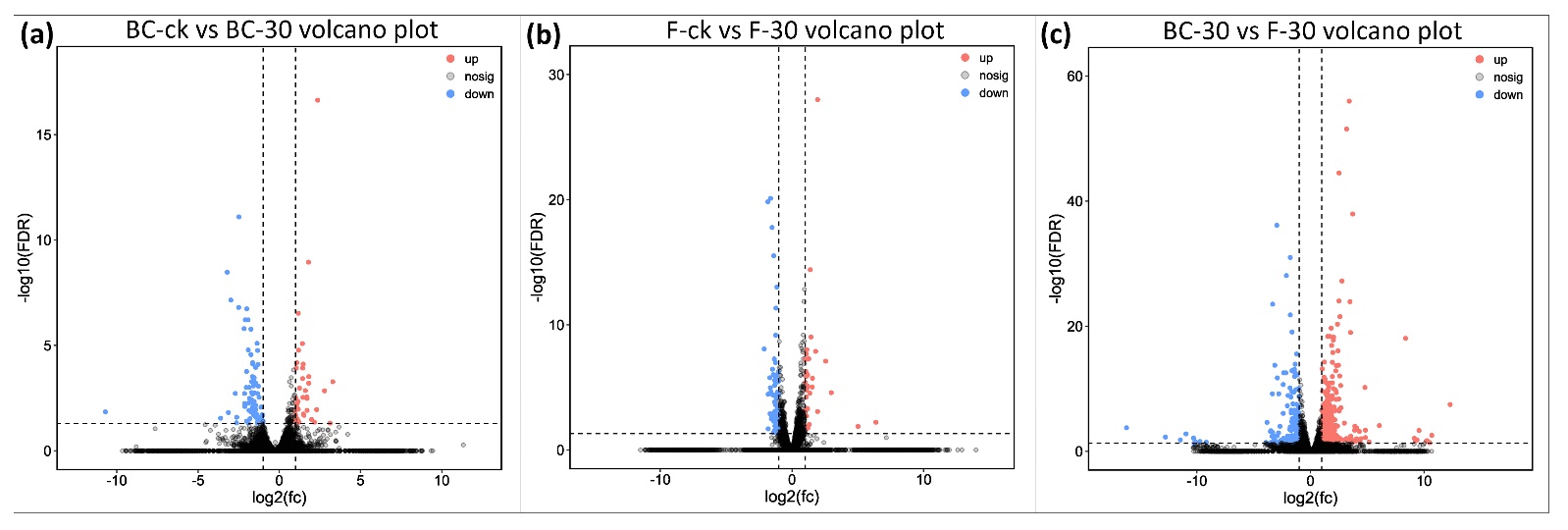
**

**Figure S1.** Differentially expressed genes after 24 h of exposure in (a) beta-cypermethrin-treated (BC-30) and control (BC-C) group. (b) fipronil-treated (F-30) and control (F-C) group. The X-axis indicates the fold change in gene expression. The dot on the X-axis indicates the statistically significant of expression of genes. On the Y-axis, -log10 (FDR) indicates P0.05. Scattered dots represnts different genes. The grey dots indicated no significant regulation, the orange dots indicated strongly up-regulated genes, and the blue dots indicated considerably down-regulated genes.

**
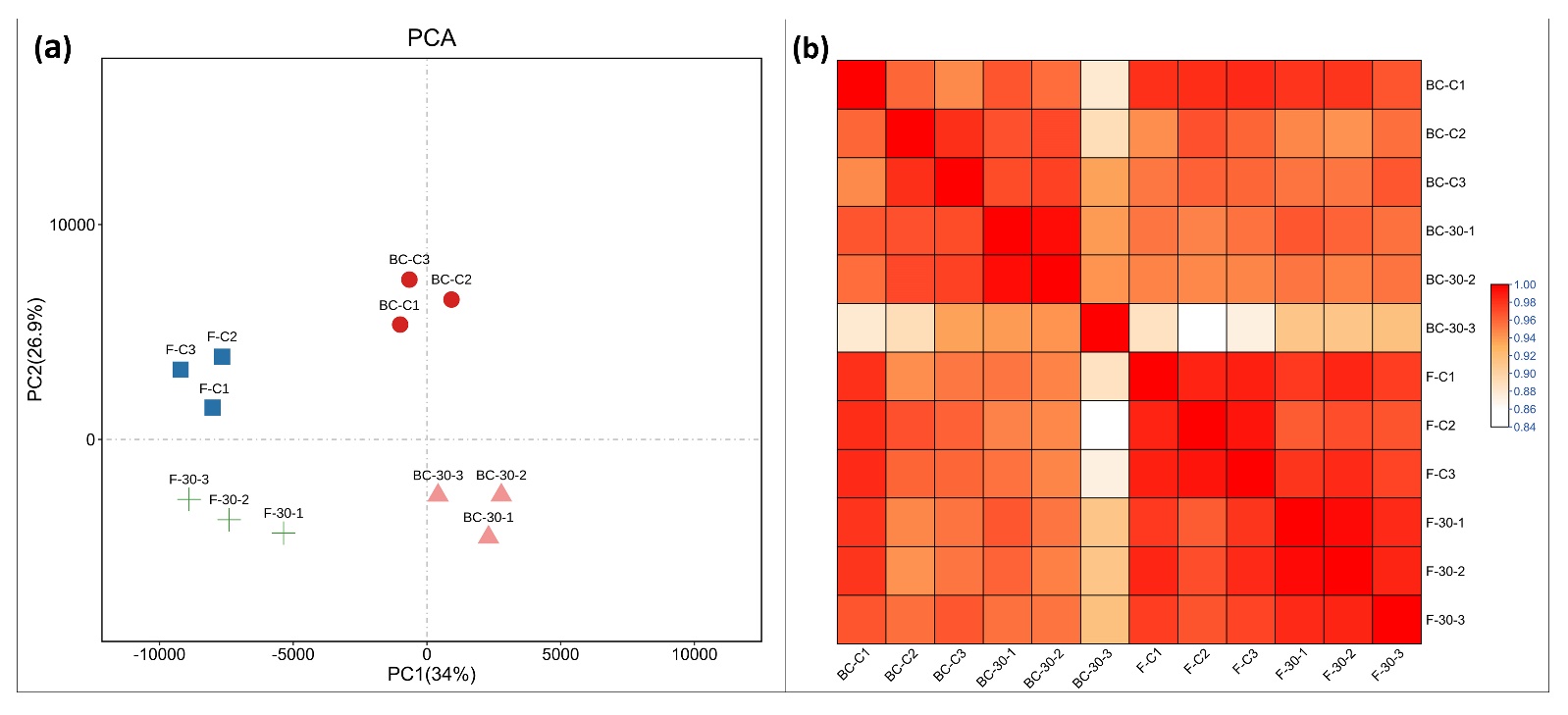
Figure S2.** Principal component analysis (PCA) and heat map of correlation analysis results of samples. (a) Principal component analysis (PCA) result. (b) Heat map of correlation analysis results of samples. The legend represents the value of the correlation coefficient, and the color changing from white to red corresponds to the correlation coefficient from small to large. The three samples of control and treatments of beta-cypermethrin and fipronil insecticide.


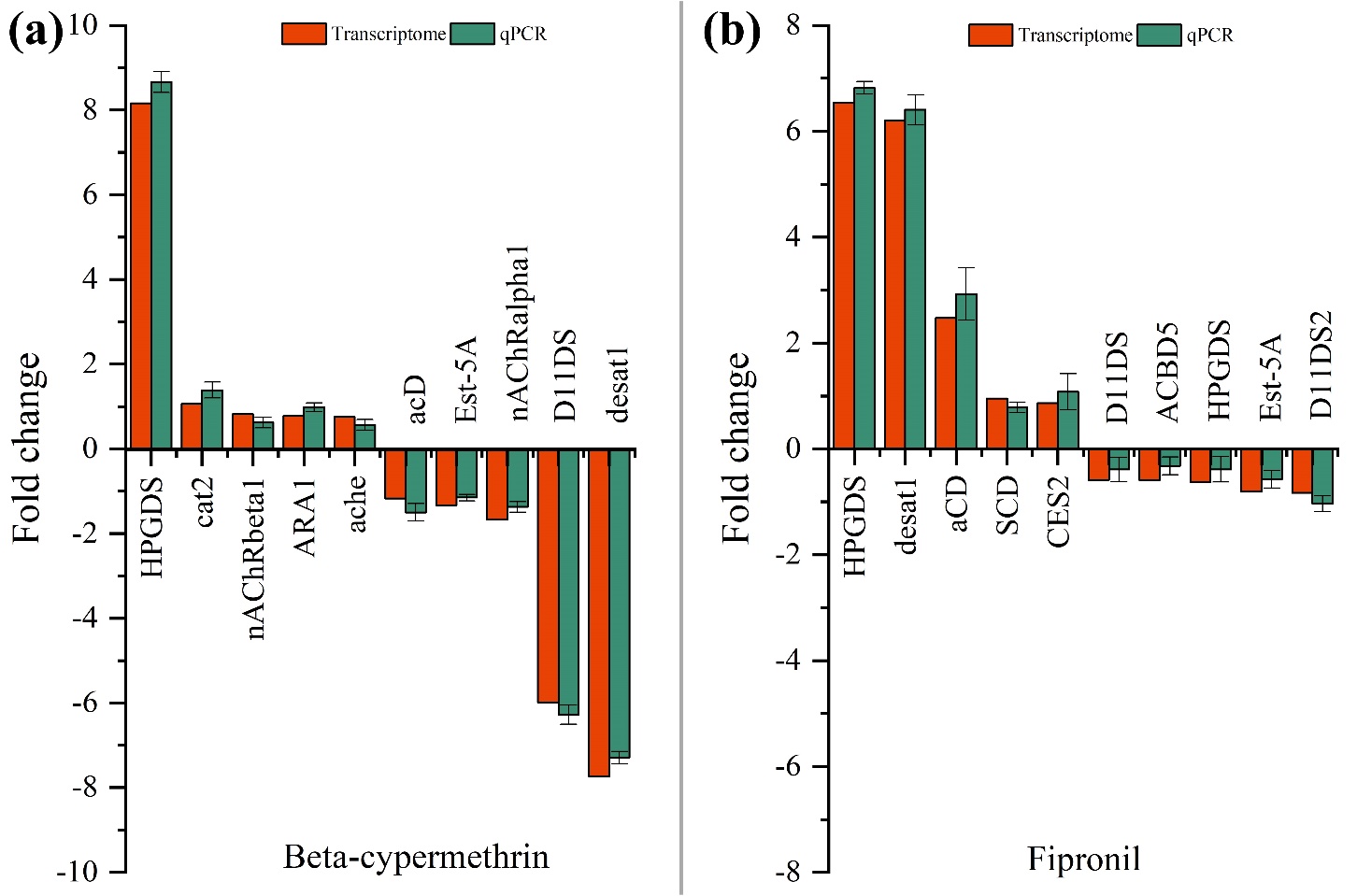
**Figure S3.** Quantitative PCR (qPCR) authentication of the DEGs associated with detoxification of both insecticides in *Solenopsis invicta* Quantitative PCR (qPCR) validation of the DEGs related to detoxification against (a) beta-cypermethrin, (b) fipronil in *Solenopsis invicta*.
